# Multimodal communication in courting fiddler crabs reveals male performance capacities

**DOI:** 10.1098/rsos.161093

**Published:** 2017-03-15

**Authors:** Sophie L. Mowles, Michael Jennions, Patricia R. Y. Backwell

**Affiliations:** 1 Animal and Environment Research Group, Faculty of Science and Technology, Anglia Ruskin University, East Road, Cambridge CB1 1PT, UK; 2 Evolution, Ecology and Genetics, Research School of Biology, The Australian National University, Canberra, Australian Capital Territory, Australia

**Keywords:** biotremology, courtship, fiddler crab, multimodal signal, stamina, vibrational signal

## Abstract

Courting males often perform different behavioural displays that demonstrate aspects of their quality. Male fiddler crabs, *Uca* sp., are well known for their repetitive claw-waving display during courtship. However, in some species, males produce an additional signal by rapidly stridulating their claw, creating a ‘drumming’ vibrational signal through the substrate as a female approaches, and even continue to drum once inside their burrow. Here, we show that the switch from waving to drumming might provide additional information to the female about the quality of a male, and the properties of his burrow (multiple message hypothesis). Across males there was, however, a strong positive relationship between aspects of their waving and drumming displays, suggesting that drumming adheres to some predictions of the redundant signal hypothesis for multimodal signalling. In field experiments, we show that recent courtship is associated with a significant reduction in male sprint speed, which is commensurate with an oxygen debt. Even so, males that wave and drum more vigorously than their counterparts have a higher sprint speed. Drumming appears to be an energetically costly multimodal display of quality that females should attend to when making their mate choice decisions.

## 1. Introduction

Sexual selection due to female choice has led to the evolution of elaborate male courtship displays that seem to communicate a male's quality as a mate [1,2]. Males often court by producing repetitive signals that range from sounds and physical movements, to electric pulses and vibrational signals (examples in [3]). In some species, males produce displays that use a single channel of communication, such as stridulating in crickets (e.g. [4,5]) or head-nodding in land-dwelling fish [6]. In other species, males produce ‘multimodal’ displays, which are defined as ‘composite signals received through more than one sensory channel’ [7]. For example, male golden-collared manakins (*Manacus vitellinus*) use both body movements and sounds in their courtship displays [8] and male peacock spiders (*Maratus volans*) make both distinctive movements and produce vibratory signals to court females [9,10].

Does the use of multiple courtship signals simply serve to confirm the information being communicated (the redundant signal hypothesis) (reviews: [11,12])? Or do different signals advertise different aspects of a male's quality (the multiple message hypothesis)? We might assume that sending multiple messages involves the use of different channels of communication (e.g. sound and vision), but this need not be the case. For example, male *Anolis* lizards extend their dewlaps and perform ‘push-up’ displays in territorial displays. These are both visual signals: but dewlap size is thought to advertise a male's bite force [13], while the vigour of the ‘push-up’ display is assumed to signal a male's stamina [14]. Conversely, it is possible that signals that use different channels of communication may provide the same information. For example, courtship displays often seem to be indices of stamina, and this information can be conveyed in several ways [3,15]. For instance, in some species males make vibrational signals that convey information about their stamina to females (e.g. wolf spiders that drum on leaves [16,17]); whereas in other species, the rate of acoustic advertisement calling provides females with information about a male's energetic reserves (e.g. crickets; [5]). To date, few studies have looked at species that court using several channels of communication to ask how different signal types are related to energy expenditure, or to ask about the relative investment that males put into each type of signal. Here, we address this oversight by studying courtship in the banana fiddler crab, *Uca mjoebergi*.

Fiddler crabs, genus *Uca*, are well known for their extreme sexual dimorphism. Females have two small feeding claws, while one of the claws in males is greatly enlarged (the major claw) [18]. In many fiddler crab species, males court by making a repetitive waving motion with their major claw [19]. Females prefer males with larger claws (e.g. *U. perplexa* [20]) that are waved at higher rates (e.g. *U. mjoebergi* [21]). Claw waving is energetically costly: lactic acid levels in males' haemolymph become elevated when they wave, presumably because of anaerobic respiration [22]. However, no study has directly linked a male's wave rate to associated costs. If there is a positive relationship between the vigour of waving and the accumulation of energetic costs, it would confirm that courtship waving is a signal of stamina [3]. Such energetically costly ‘signals of stamina’ are likely to be important to females as they would reflect the ability of the male to perform other demanding activities such as sprinting to avoid predators, while also indicating that the male has accrued sufficient energy reserves to expend in display, thus demonstrating that he is an effective forager. These are attributes with which a choosy female should want to provision her offspring [23]. Further, the ability to perform a demanding display well may also indicate that the male is in good condition, thus preventing females from mating with diseased or parasitized males [24].

In some fiddler crab species, males additionally produce vibrational signals via rapid movements of their major claw, which are transmitted through the ground, despite the stridulating major claw making little, if no contact with the substrate [25–27]. Males perform this ‘drumming’ [25] vibrational display as a female approaches the male's burrow [26] and even continue to drum after she follows him into the burrow. There is evidence that females prefer males that emit rapid vibrations [28], but the specific function of drumming has yet to be investigated. Drumming may contain information not provided by the waving display (i.e. the multiple message hypothesis). For example, the vibratory nature of the display might advertise structural properties of a male's burrow. Burrow dimensions are important to a female because she stays inside the mated male's burrow to incubate her eggs (see [28]). Burrow properties affect the speed at which eggs develop into larvae, which is crucial to ensure the correct timing of larval release during a nocturnal spring tide (e.g. [29,30]). Thus, drumming might allow females to choose a male with an appropriate burrow if the acoustic properties of the drumming display permit the female to assess the physical characteristics of the burrow. Alternatively, drumming might advertise other aspects of male quality, such as size (multiple message hypothesis). Larger males are likely to produce more powerful drums, as occurs with shell-rapping by hermit crabs [31]. If so, drumming might be an index of male size rather than an inherently costly handicap signal [32], although indices and handicaps are often hard to distinguish [33] (e.g. drumming with a larger claw might consume more energy). Finally, drumming might reinforce information already provided by waving (redundant signal hypothesis). Vibrational displays in other taxa are energetically costly (e.g. wolf spiders [16]). Drumming by a male fiddler crab might augment his claw-waving display by confirming his ability to perform a demanding activity: consistent with a signal of stamina.

In this study we examined signalling by male fiddler crabs in the final stage of courtship as a female approaches a male's burrow. We specifically tested the energetic consequences of waving and drumming with *in situ* performance (sprint speed) trials to test whether fine-scale aspects of waving and/or drumming predict, hence signal, post-display performance. We also tested whether the acoustic properties of the drumming display better agreed with the multiple messages or redundant signal hypotheses.

## 2. Material and methods

### 2.1. Study site and organisms

We carried out fieldwork on *U. mjoebergi* from September to November 2014 at East Point Reserve, Darwin, Australia, during the diurnal low tide period of neap tides. In our experiments, we presented each treatment male with a different stimulus female (see below). We always chose males (control and treatment) that we had earlier seen waving, as they were more likely to court a stimulus female if one was presented (*N* = 89). Males were chosen haphazardly with respect to size to reflect the natural size range (carapace widths: treatment males: 11.81 ± 0.10 mm, control males: 11.85 ± 0.09 mm). (All summary data are presented as mean ± s.e.).

### 2.2. Stimulus females

We captured resident females at their burrows. Each female was then carefully tethered to the end of a thin wooden dowel (2 mm diameter), which was attached to a long dowel (1 cm diameter, 120 cm in length). The stimulus female was then moved towards a focal male to elicit natural courtship behaviour. These females were all similar in size (carapace width: 9.15 ± 0.07 mm) to reduce any effect of variation in female body size on male courtship effort.

### 2.3. Behavioural experiments

Once we identified a focal male, we placed a contact microphone (TWA-3S, Japan; frequency response 100 Hz–8 kHz/±3 dB) 1.5 cm from the burrow entrance. We then drew a series of faint lines in the sand at 10 cm, 5 cm and 2.5 cm from the burrow to provide set distances at which to temporarily halt the female as we moved her towards the male. We used a Sony Handicam DCR-SR45E to film both the male's behaviour and the female's progress along the graduated track. To start a trial, the stimulus female was held at the 10 cm mark for 1 min, after which she was moved to the 5 cm mark for 1 min, then to the 2.5 cm mark for 1 min and finally to the burrow entrance for one minute. Throughout her approach, we video recorded the male's behaviour and recorded his drumming display as 24 bit WAV files using a Tascam, Linear PCM Recorder (DR-07 Mk II) receiving input from the contact microphone. Both the contact microphone and the recorder were calibrated at the start of each trial to receive and record at 50% maximum on each occasion to prevent peaking of the microphone.

### 2.4. Sprint performance experiments

Immediately after the trial, we captured the male and released him at the start of a 1.5 m long sprint track (made of two vertical plastic sheets that were 5 cm apart and embedded in the substrate (see electronic supplementary material, S1). Males ran spontaneously when released and were pursued along the racetrack with a wooden probe. The sprint speed over 50 cm was recorded from a ‘start line’ to a ‘finish line’ in the first half of the track. However, the track was necessarily longer than this distance to ensure that the crab ran at full speed over the required distance instead of slowing towards the end of the 50 cm. Control males were tested identically, except that they were not exposed to an approaching stimulus female. We also ensured that, as with treatment males, we had earlier seen control males waving. After the sprint trial, the focal male was placed in an isolated cup (10 cm diameter) filled to a depth of 1 cm with seawater, and left in the shade for an hour before being retested. This process was repeated twice so that we eventually had three sprint speed measures per male.

### 2.5. Crab morphology

After the final sprint trial, we measured the male's carapace width and major claw length to the nearest 0.1 mm using a pair of dial callipers. We also noted his handedness and if his claw was regenerated or original. Only five males per treatment group had a regenerated claw.

### 2.6. Measuring burrow parameters

After the burrow owner had been captured, we surrounded the burrow entrance with a plastic collar to prevent other crabs from entering it. We then filled the burrow with Polyfilla Expanding Foam. We allowed the cast to set for an hour before digging it out, cleaning it and allowing it to dry (electronic supplementary material, S2). We measured the burrow volume by submerging the cast in a 1 l measuring cylinder to record how much water was displaced (to the nearest 2.5 cm^3^).

### 2.7. Behavioural analyses

We combined the video and audio files for each encounter using Windows Live Movie Maker. The audio from the video files was muted to make any drumming recorded by the contact microphone more salient. We then watched the combined video and audio files and scored male behaviours using jWatcher event recording software. We recorded each claw wave and each bout of drumming (and noted if it was on the substrate, at the burrow entrance, or underground).

### 2.8. Acoustic analyses

The frequencies recorded from the drumming crabs (344.5–728.82 Hz) were well within the frequency characteristics of the contact microphone (TWA-3S, Japan; frequency response 100 Hz–8 kHz/±3 dB) and that expected for a sand substrate [28,34].

We analysed audio files using Raven Pro 1.4. The spectrograms were used to identify each individual signal component making up each bout of drumming (see electronic supplementary material, S3), from which the peak frequency (the frequency (hertz) at which the highest amplitude occurred) and peak power (decibel) could be extracted. We then tested whether drumming behaviour changed when the male was on the substrate (and with the distance of the female from the male), at the burrow entrance, or within the burrow. Any change in drumming allows us to identify whether drumming advertises male or burrow characteristics. For example, changes in vigour would be indicative of a display of male stamina, whereas a more consistent signal structure may indicate that the drumming signal is used to advertise characteristics other than the performance capacity of the male, such as the physical structure of the burrow. Further, the precise point at which each individual beat was produced was recorded, enabling us to make fine-scale analyses of signal component production and escalation patterns.

### 2.9. Quantifying signal escalation

We measured the bivariate correlations between the vigour of signal production and the point during the trial at which the signal element was produced. Thus, the interval between bouts of drumming; the interval between individual ‘beats’; and the number of beats/bout were each correlated against when they were produced during the trial. For the interval measures, the signal is escalated if there is a negative correlation (i.e. intervals are shorter as the interaction progresses). For the beats/bout the signal is escalated if there is a positive correlation (i.e. more beats are produced per bout as the interaction progresses). Conversely, signal reduction occurs if the correlations are in the opposite directions. Here, we assign discrete categories to the type of signal change (escalation, de-escalation or static) depending on whether the correlation is significantly greater than zero, significantly less than zero or non-significant [3,5].

### 2.10. Statistical methods

To test for differences in the number of waves or drums produced when the female was at different distances from the male (10, 5, 2.5 or 0 cm from the burrow), we used Friedman's rank sum test because there were four repeated measurements per male.

To assess whether courtship is energetically demanding, we compared the performance (i.e. sprint speed) of treatment males (that had just courted) and control males (that had not courted). Sprint speed was log_10_ transformed to meet the requirements of the parametric tests. We used a repeated measures ANOVA because we had three sprint estimates per male. The between subject factor was male status (courtship treatment or control) and the repeated measure was the trial: Trial 1 (immediately after capture), Trial 2 (1 h after capture) and Trial 3 (2 h after capture). *Post hoc* analyses were conducted using one-way ANOVAs with male status as the factor.

We calculated the relationships between male performance, indices of drumming vigour (mean bout interval, mean beat interval and beats/bout), morphology and drumming frequency (hertz) using Pearson's correlations. We quantified the relationships between drumming amplitude, morphology and burrow volume using Spearman's correlations as amplitude and burrow volume could not be normalized by transformation. All data were analysed using R v. 3.1.0.

## 3. Results

### 3.1. Male courtship sequence

**Figure 1.**
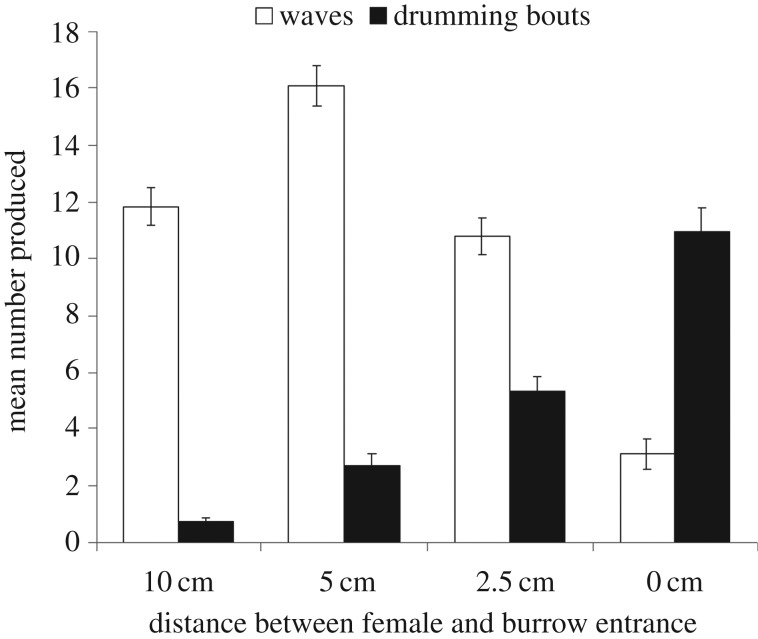
The mean number of waves and bouts of drumming produced by a male when the female is 10 cm, 5 cm, 2.5 cm and 0 cm from his burrow entrance. Error bars represent standard errors.

As a female approached a male his waving decreased significantly (Friedman: $\chi_{3}^{2}=137.445,$ *p* < 0.001), while the number of bouts of drumming increased significantly (Friedman: $\chi_{3}^{2}=139.035,$ *p* < 0.001; figure 1). Across males there was a significant positive relationship between the total number of waves and the number of bouts of drumming performed (*r* = 0.265, d.f. = 87, *p* = 0.012).

### 3.2. Courtship and sprint performance

**Figure 2.**
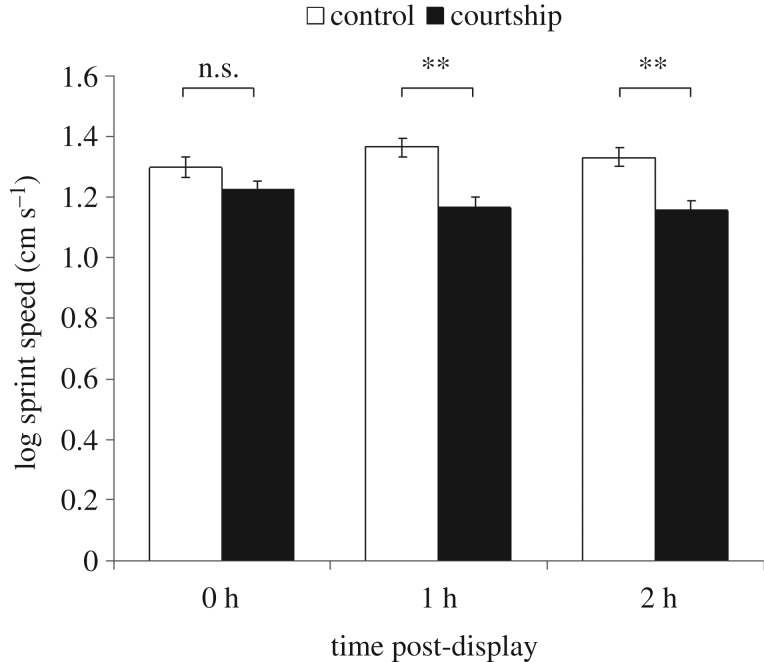
The performance capacities (sprint speeds over 50 cm) of males that had courted and control males 2 h, 1 h or immediately after displaying. Error bars represent standard errors.

Control males had significantly higher sprint speeds than males that had just courted (*F*_1,290_ = 16.567, *p* < 0.001; figure 2). There was, however, a significant difference between the two types of males in how sprint speed changed over the three trials (*F*_2,290_ = 4.247, *p* = 0.015). There was no difference in sprint speed between control and recently courting males immediately after capture (Trial 1: *F*_1,145_ = 2.616, *p* = 0.108). However, 1 or 2 h later recently courting males sprinted significantly more slowly than control males (Trial 2: *F*_1,145_ = 18.06, *p* < 0.001; Trial 3: *F*_1,145_ = 14.84, *p* < 0.001).

### 3.3. Sprint performance and courtship vigour

**Figure 3.**
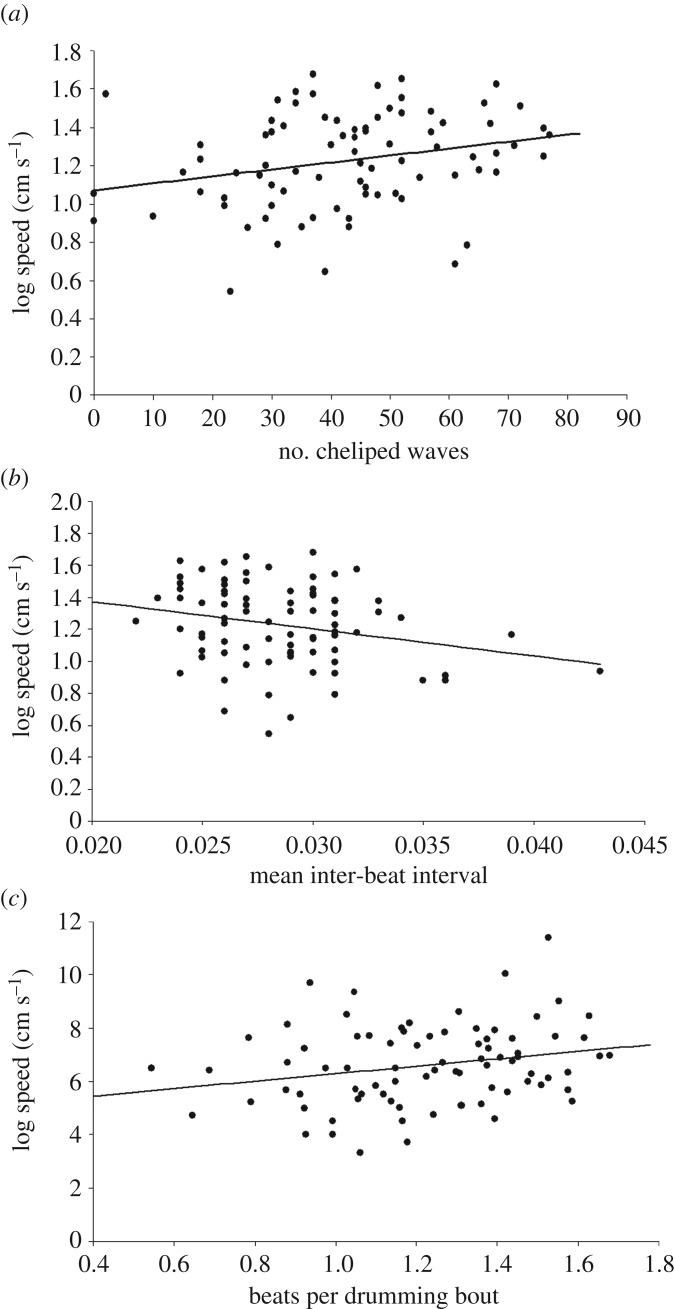
The relationships between a male's performance capacity (sprint speed in cm s^−1^) immediately after displaying and (*a*) the number of cheliped waves (*R*^2^ = 0.065), (*b*) the mean interval between cheliped beats (*R*^2^ = 0.054) and (*c*) the mean number of cheliped beats per bout of drumming (*R*^2^ = 0.055).

In the first performance trial, males that produced more claw waves, males that had a shorter interval between drum beats and males that performed more beats per bout ran significantly faster (figure 3). There was, however, no relationship between the number of drumming bouts, or the interval between these bouts, and how fast the male sprinted. In the second and third performance trials, none of the five measures of courtship vigour were related to a male's sprint speed (table 1).

**Table 1.** The relationship between courtship display vigour and performance in subsequent sprint trials. Performance was measured as sprint speed in cm s^−1^. Significant results are indicated in bold typeface.

	*R*	d.f.	*p-*value
mean number of waves			
performance immediately post-display	0.255	80	**0.021**
performance 1 h post-display	0.028	80	0.802
performance 2 h post-display	0.120	80	0.281
mean number of drumming bouts			
performance immediately post-display	0.161	80	0.150
performance 1 h post-display	−0.047	80	0.677
performance 2 h post-display	0.167	80	0.133
*drumming characteristics*			
mean inter-drumming bout interval			
performance immediately post-display	−0.147	79	0.190
performance 1 h post-display	0.196	79	0.079
performance 2 h post-display	0.140	79	0.214
mean inter-beat interval			
performance immediately post-display	−0.243	79	**0.029**
performance 1 h post-display	0.035	79	0.756
performance 2 h post-display	−0.074	79	0.514
mean beats per bout			
performance immediately post-display	0.235	79	**0.034**
performance 1 h post-display	0.087	79	0.439
performance 2 h post-display	−0.052	79	0.646

### 3.4. Sprint performance and male size

For males that had recently been induced to court, larger individuals ran significantly faster in all three performance trials. By contrast, for control males, there was no relationship between their size and sprint speed in any of the three performance trials (table 2).

**Table 2.** The relationship between male carapace width and performance in subsequent sprint trials. Performance was measured as sprint speed in cm s^−1^.

	*r*	d.f.	*p*-value
courtship trials			
performance immediately post-display	0.247	80	0.025
performance 1 h post-display	0.301	80	0.006
performance 2 h post-display	0.226	80	0.042
control trials			
performance immediately post-display	0.171	63	0.173
performance 1 h post-display	−0.011	63	0.932
performance 2 h post-display	−0.025	63	0.843

### 3.5. Male size and courtship vigour

Larger males performed significantly more beats per bout of drumming. There were, however, no significant relationships between male size and any of the other four measures of courtship vigour (table 3).

**Table 3.** The relationship between male carapace width and indices of display vigour. Significant results are indicated in bold typeface.

	*r*	d.f.	*p*-value
mean number of waves	0.124	87	0.246
mean number of drumming bouts	−0.112	87	0.297
mean inter-drumming bout interval	0.040	85	0.713
mean inter-beat interval	−0.048	85	0.656
mean beats per bout	0.294	85	**0.006**

### 3.6. Courtship escalation and sprint performance

Males switch from waving to drumming as a female approaches (figure 1). Consequently, waving always declines while drumming escalates. We, therefore, investigated net courtship escalation by combining data on the intervals between claw waves and bouts of drumming. We then compared the performance of three types of males; those that showed an escalation (*N* = 16), de-escalation (*N* = 20) or a static rate of courtship display (*N* = 51) as the female approached. To do this we carried out generalized linear models (GLMs) using a binomial error distribution and logit link function to test whether the category of overall signal production exhibited by the male (escalation, de-escalation or static rate of display) predicted their subsequent sprint speeds in the performance capacity trials. Thus, signal escalation was the factor in the models, while sprint speed in each trial was the dependent variable. As crab size is related to sprint performance, we included it as a covariate in our models.

In the initial performance trial there was no interaction between crab size and male performance so the interaction was removed from this model. There was no significant difference in sprint speed between the three types of males (GLM: *z* = 1.442, d.f. = 77, *p* = 0.149) and no overall relationship between sprint speed and male size (GLM: *z* = 1.129, d.f. = 77, *p* = 0.259).

**Figure 4.**
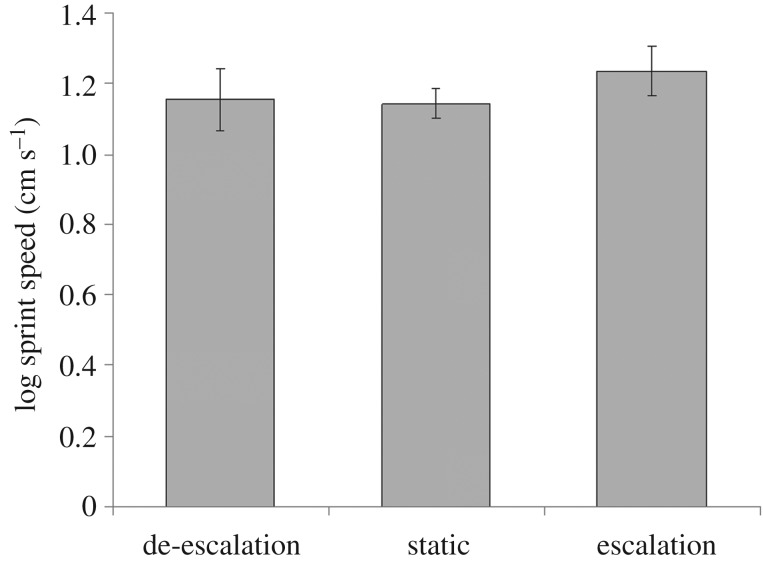
The mean performance capacities 1 h post-display according to whether males escalated, de-escalated or maintained a static rate of signalling.

**Figure 5.**
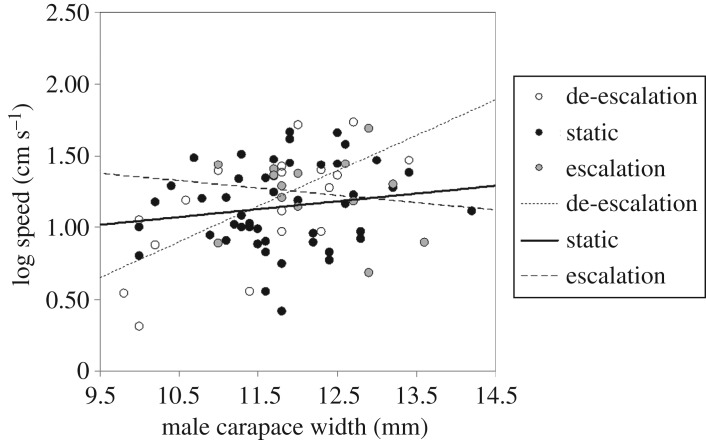
The interaction between male carapace width, performance capacity and whether a male escalated, de-escalated or maintained a static rate of signalling.

For the second and third performance trials (1 and 2 h later), there were significant differences in sprint speed between the three types of males (GLM: Trial 2: *z* = 2.199, *p* = 0.028; Trial 3: *z* = 1.990, *p* = 0.047; both d.f. = 76, figure 4). There was also a difference among male types in the relationship between body size and sprint speed (GLM: Trial 2: *z* = −2.255, *p* = 0.024; Trial 3: *z* = −1.992, *p* = 0.046; both d.f. = 76; figure 5). Among males that reduced their courtship display vigour, larger males sprinted faster (Trial 2: *r* = 0.664, *p* = 0.002; Trial 3: *r* = 0.502, *p* = 0.028; both d.f. = 17). There was no such relationship for males that did not change their courtship display vigour (*r* = 0.173, *p* = 0.235; *r* = 0.177, *p* = 0.223; both d.f. = 47), or for those that escalated their display vigour (Trial 2: *r* = −0.152, *p* = 0.603; Trial 3: *r* = −0.156, *p* = 0.592; both d.f. = 12).

### 3.7. Escalation in the rate of drumming

Most males maintained a static rate of drumming as courtship progressed (table 4). Other males escalated their display by shortening the intervals between bouts, or between beats within a bout. Very few males (less than 3%) increased the intervals between bouts, or between beats within a bout. Finally, for most males the number of drums per bout remained constant as a female approached, although 15% of males (*N* = 13) did reduce their rate of drumming.

**Table 4.** The number of males producing drumming at constant, escalating and de-escalating rates.

	static	escalation	de-escalation
bout interval	54	28	2
beat interval	45	39	3
drums per bout	69	5	13

### 3.8. Acoustic properties of drumming and male size

**Figure 6.**
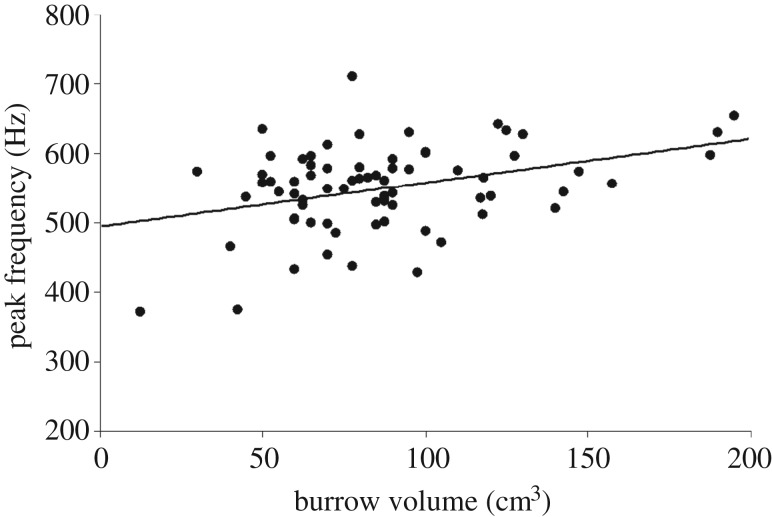
The relationship between burrow volume and the peak frequency of cheliped drumming.

Larger males did not drum at a higher frequency (hertz) or amplitude (decibel) (table 5). Larger males constructed larger volume burrows (*r*_s_ = 0.452, *N* = 89, *p* < 0.001). The peak frequency (hertz) of drumming from within the burrow was higher for males with larger burrows (table 6, figure 6), and this remained the case even after controlling for male size (partial correlation, *r* = 0.352, *N* = 72, *p* = 0.003). There was, however, no relationship between burrow volume and the amplitude (decibel) of drumming. Finally, males with larger burrows produced more drums per bout (*r* = 0.245, d.f. = 85, *p* = 0.022), although this was driven by larger males producing more drums per bout (partial correlation, *r* = 0.219, *N* = 87, *p* = 0.043) rather than burrow volume being directly linked to drumming vigour (partial correlation, *r* = 0.143, *N* = 87, *p* = 0.189).

**Table 5.** Correlations of male carapace width against the peak frequency and amplitude of drumming.

frequency of drumming (Hz)	range	mean	*r*	d.f.	*p*-value
surface	344.5–728.82	537.86	−0.142	35	0.403
entrance	367.23–700.58	540.65	−0.051	81	0.645
underground	370.60–711.03	549.61	0.130	70	0.277

amplitude of drumming (dB)			*r*_s_	*N*	*p*-value
surface	61.35–131.86	80.10	0.033	37	0.846
entrance	72.71–142.85	90.61	−0.120	83	0.279
underground	77.59–140.94	94.36	−0.035	72	0.771

**Table 6.** The relationship between burrow volume and the peak frequency or amplitude of drumming. Significant results are indicated in bold typeface.

	*r*_s_	*N*	*p*-value
frequency of drumming			
surface	−0.190	37	0.261
entrance	0.112	83	0.312
underground	0.273	72	**0.020**
amplitude of drumming			
surface	−0.112	37	0.511
entrance	−0.182	83	0.099
underground	−0.132	72	0.268

## 4. Discussion

In *U. mjoebergi*, males initially court a female by waving their brightly coloured major claw. As she approaches they switch to stridulating their claw, producing a ‘drumming’ signal which, although involving little to no contact between the major chela and the substrate, is transmitted through the substrate as a series of rapid vibrations. Wave rate influences female choice: females prefer males that wave at a higher rate [21]. Females also prefer males with a larger claw [20]. We do not yet know if female choice in *U. mjoebergi* is influenced by drumming, but females in another *Uca* species select males based on the vigour of their drumming display [28]. Here we confirm that drumming in *U. mjoebergi* conveys information about both a male's stamina and the size of his burrow, suggesting that females would benefit by choosing males using drumming signals.

The simultaneous production of repetitive visual displays and vibrational courtship signals occurs in several invertebrate taxa (e.g. Saliticid [9,10] and Lycosid [16,17] spiders). In fiddler crabs, however, the production of vibrational signals (drumming) can occur independently from visual displays (claw waving). This occurs when a male is underground in his burrow but the female is still on the surface (e.g. [26]). Similarly, in some spiders, such as tarantulas (*Eupalestrus weijenberghi* and *Acanthoscurria suina*), males on the surface drum to send vibrational signals to females that are underground [35]. In *U. mjoebergi*, there is a gradual shift from waving to drumming as courtship progresses (electronic supplementary material, video). We showed that males that wave at a high rate also drum at a high rate. This suggests that the vibrational signal produced by drumming serves a similar function to that of the waving display, consistent with the redundant signal hypothesis [11,12]. The advantage of using two modes of communication is, however, that males can still provide information from inside their burrow when they are no longer visible to a female.

Repetitive courtship displays can advertise the quality of a male by: (i) allowing the female repeated opportunities to assess each individual signal and correct for production/reception errors (i.e. to increase the accuracy of information acquisition; see [3,15]); (ii) allowing the male to demonstrate his ability to bear signalling costs, which might be extrinsic (e.g. enhanced predation risk, see [36,37]), or intrinsic (e.g. energetic costs of displaying [5,16,22]). We found that male *U. mjoebergi* suffer energetic costs when courting. Males experimentally induced to court had a significantly poorer performance in sprint trials than non-courting control males. However, this difference was initially absent, and it only became apparent 1 or 2 h after courting. This suggests that the build-up of lactic acid associated with signalling in fiddler crabs [22] reduces future performance, which is commensurate with an ongoing oxygen debt. The seemingly heavy investment required to drum and wave is likely to allow females to select physically fit mates.

Female *U. mjoebergi* can select physically fit males if they pay attention to both the absolute level of courtship signal production, and whether or not it increases as they approach a male. In the initial performance capacity trials immediately post-courtship, before the effects of oxygen debt were apparent, males that had more vigorous displays (e.g. more waves, shorter intervals between vibrational signals, and more signals per drumming bout) had a higher performance capacity (i.e. sprint speed). Furthermore, when retested 1 h later, males that escalated their rate of courtship during a female's approach had a higher sprint speed than males whose display rate stayed the same or declined.

The demanding courtship display of male fiddler crabs is informative as males seem to vary considerably in quality independently of body size. A broad range of performance capacities is evidenced by the way in which sprint capacity in the later performance trials is affected by an interaction between male size and the change in courtship rate. Males that reduced their display rate exhibited a significant positive correlation between speed and carapace width, whereas this relationship was absent in males that either escalated or maintained a static display rate. Once males have engaged in an energetically demanding display, such that the anaerobic threshold is reached (most likely to have occurred for males that reduced their courtship, an indication of exhaustion in signalling animals), body size is the best predictor of performance, whereas initially stamina is highly variable amongst individuals and not predicted by body size. Indeed, Matsumasa & Murai [22] demonstrated great variation in baseline levels of haemolymph glucose in fiddler crabs, confirming that there can be significant variation in male quality in this taxon. Our results supports the general finding that courtship displays are energetically costly and that females can profitably use them during mate choice to gauge male performance capacity.

Aside from being linked to a male's performance capacity, vibrational signals produced by drumming provide information about a male's burrow. Using this information could benefit females because it reduces their risk of being coerced into mating that arises when they enter a male's burrow to directly assess its volume [38]. In *U. mjoebergi*, the amplitude (decibel) of vibrational signals was unrelated to a male's size or to that of his burrow, however, the peak frequency (hertz) was positively related to burrow volume. This is consistent with findings for substrate-borne signals in insects [39] and arachnids [40]: their frequency is not dependent on the signaller's body size (but see [28]), but instead is governed by the properties of the substrate [41]. In *U. mjoebergi*, larger volume burrows have proportionately wider openings, usually having been created by crabs of larger carapace widths. Thus, the higher frequencies produced from larger burrows are not due to narrower openings. However, larger volume burrows are longer and deeper, presenting a greater length of sand through which the low frequency components may be filtered out during their transmission. This is an area that requires further research to fully resolve, including an investigation of the physical properties of the substrate (e.g. compactness and saturation). In *U. mjoebergi*, we also showed that larger males produce more signal components (beats) per bout of drumming, and that this is correlated with burrow volume via male size. Thus, the frequency of drumming is another signal that females can use to estimate burrow volume.

Although females generally prefer bigger males [20], the value of a larger burrow depends on the ambient temperature [42] and the stage of the lunar cycle at which mating occurs [43]. Female *U. mjoebergi* exhibit variation in their preference for male size, hence burrow volume (as the two are correlated), over the 9-day semi-lunar mating period [30]. It might, therefore, sometimes be detrimental for a male to provide additional information about burrow volume by drumming. Given that males perform drumming interspersed with waving on the substrate, but continue to drum once inside their burrow, we suggest that drumming primarily reinforces a female's perception of a male's stamina. Drumming provides an alternative means of assessment of stamina once a male has disappeared from view and waving is no longer possible. We therefore suggest that, while the hypotheses are not mutually exclusive, drumming is more consistent with the redundant signal than the multiple message hypothesis. This conclusion is further supported by the positive correlation between waving and drumming: production of both signals is seemingly governed by the same energetic mechanisms. Parallel explanations for the use of multiple signals might be common across many taxa, including those that use single (e.g. [44,45]) or multiple channels of communication (e.g. [9,46]). That is, multiple signals are implemented to reinforce a single aspect of sender quality.

## Supplementary Material

ESM 1. The in-field sprint track set up at East Point Reserve, Darwin, Northern Territory, Australia

## Supplementary Material

ESM 2. Burrow casts of male fiddler crabs.

## Supplementary Material

ESM 3. Oscillogram and spectrogram of a single drumming bout.

## Supplementary Material

ESM 4. Supporting data.

## Data accessibility

The dataset supporting this article has been uploaded as part of the electronic supplementary material.
