# Supplementary figures and images for: Multimodal communication in courting fiddler crabs reveals male performance capacities
Source: R Soc Open Sci. 2017 Mar 15;4(3):161093. doi: 10.1098/rsos.161093 (PMC5383853; doi:10.1098/rsos.161093)

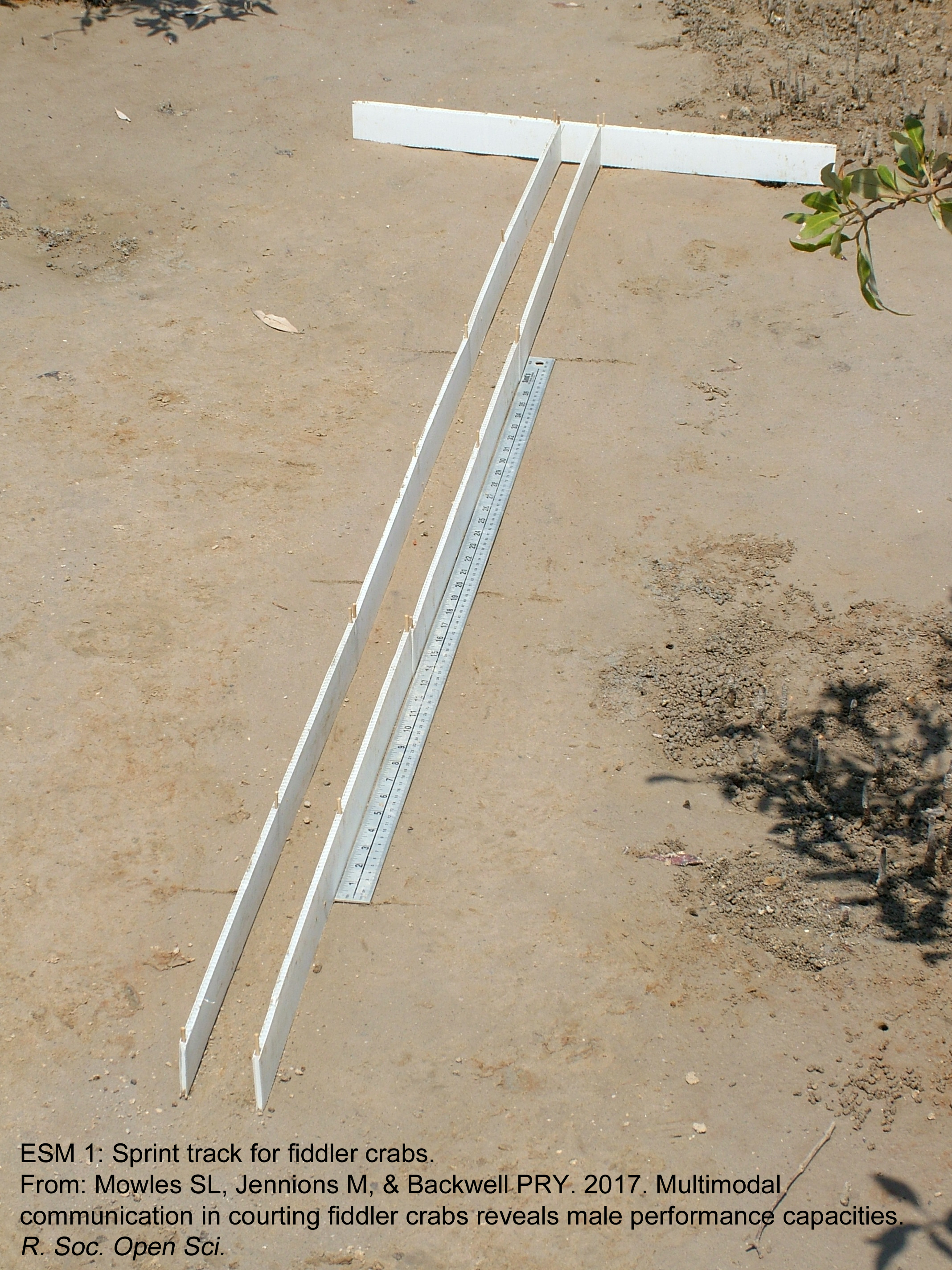

Supplement: ESM 1. The in-field sprint track set up at East Point Reserve, Darwin, Northern Territory, Australia [file rsos161093supp1.tif]

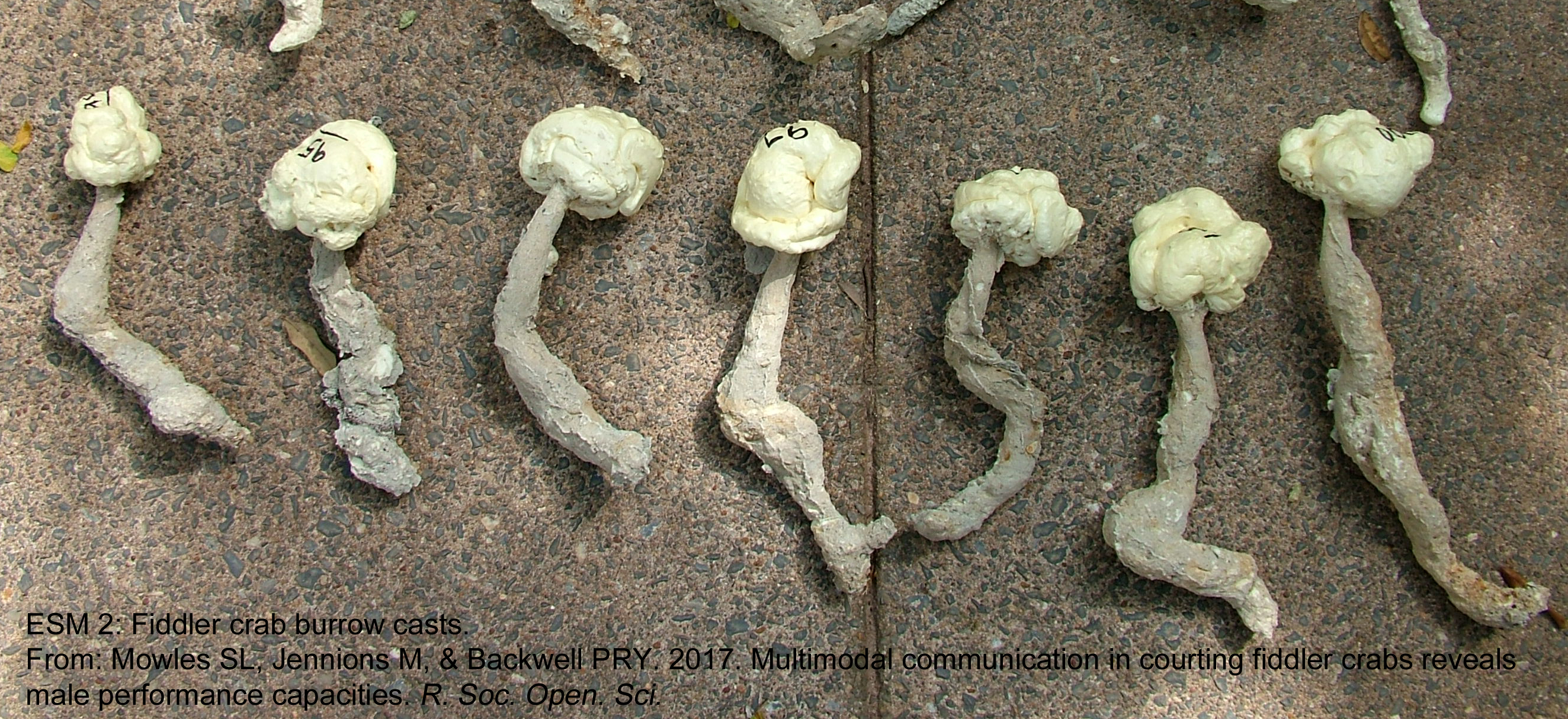

Supplement: ESM 2. Burrow casts of male fiddler crabs. [file rsos161093supp2.tif]

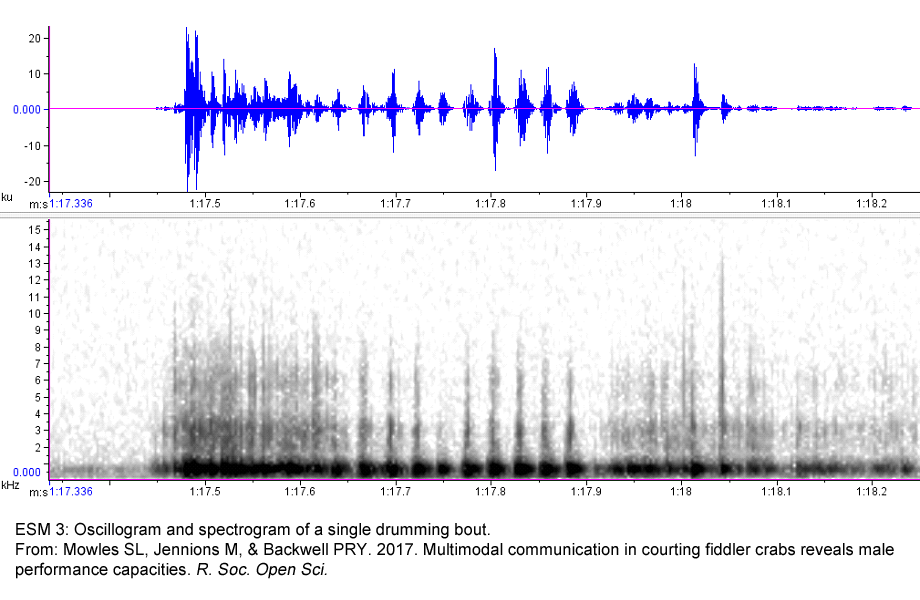

Supplement: ESM 3. Oscillogram and spectrogram of a single drumming bout. [file rsos161093supp3.tif]
